# Artificial intelligence-driven systems engineering for next-generation plant-derived biopharmaceuticals

**DOI:** 10.3389/fpls.2023.1252166

**Published:** 2023-11-15

**Authors:** Subramanian Parthiban, Thandarvalli Vijeesh, Thashanamoorthi Gayathri, Balamurugan Shanmugaraj, Ashutosh Sharma, Ramalingam Sathishkumar

**Affiliations:** ^1^ Plant Genetic Engineering Laboratory, Department of Biotechnology, Bharathiar University, Coimbatore, India; ^2^ Tecnologico de Monterrey, School of Engineering and Sciences, Centre of Bioengineering, Queretaro, Mexico

**Keywords:** artificial intelligence, molecular pharming, synthetic biology, deep learning, machine learning

## Abstract

Recombinant biopharmaceuticals including antigens, antibodies, hormones, cytokines, single-chain variable fragments, and peptides have been used as vaccines, diagnostics and therapeutics. Plant molecular pharming is a robust platform that uses plants as an expression system to produce simple and complex recombinant biopharmaceuticals on a large scale. Plant system has several advantages over other host systems such as humanized expression, glycosylation, scalability, reduced risk of human or animal pathogenic contaminants, rapid and cost-effective production. Despite many advantages, the expression of recombinant proteins in plant system is hindered by some factors such as non-human post-translational modifications, protein misfolding, conformation changes and instability. Artificial intelligence (AI) plays a vital role in various fields of biotechnology and in the aspect of plant molecular pharming, a significant increase in yield and stability can be achieved with the intervention of AI-based multi-approach to overcome the hindrance factors. Current limitations of plant-based recombinant biopharmaceutical production can be circumvented with the aid of synthetic biology tools and AI algorithms in plant-based glycan engineering for protein folding, stability, viability, catalytic activity and organelle targeting. The AI models, including but not limited to, neural network, support vector machines, linear regression, Gaussian process and regressor ensemble, work by predicting the training and experimental data sets to design and validate the protein structures thereby optimizing properties such as thermostability, catalytic activity, antibody affinity, and protein folding. This review focuses on, integrating systems engineering approaches and AI-based machine learning and deep learning algorithms in protein engineering and host engineering to augment protein production in plant systems to meet the ever-expanding therapeutics market.

## 1 Introduction

Plant molecular pharming refers to the recombinant expression of biologics including vaccines, hormones, therapeutics and diagnostic reagents in plant-based systems. The field is gaining attention since the biologics produced from plants are efficient and similar to products from other conventional systems with the advantage of eukaryotic host performing post-translational modifications. Some of these recombinant biologics produced in plant systems are SARS-CoV2 virus-like particle (VLPs), spike antigen, anti-SARS-CoV2 mAb H4 and B38, anti-EBV (Ebola virus) mAb 6D8, 4H2 IgG and IgM (against *Coccidioides*), antimicrobial peptide (AMP) LL-37 and human apolipoprotein A-I_Milano_ (Apo A-I_Milano_) (Fulton et al., 2015; Holásková et al., 2018; Ali and Kim, 2019; Shanmugaraj et al., 2020; Jugler et al., 2022; Zhao et al., 2023). Various model plant systems have been used as stable or transient heterologous expression hosts for recombinant protein production that include, tobacco (*Nicotiana benthamiana* and *Nicotiana tabacum*), Arabidopsis, tomato, potato, rice, maize, soybean, etc. (Ghag et al., 2021; Lobato Gómez et al., 2021). The plant host systems are useful in many aspects such as cost-effectiveness, multimeric protein assembly, scale-up and safety (minimal/no risk of human pathogen contaminations). Even with the listed advantages, there are few limitations to use plants as expression systems such as lack of humanized *N*-glycosylation post-translational modification which is needed for antibody production and stability of plant-produced proteins are still a concern (Sethi et al., 2021). Recombinant biologics production is dependent on several factors such as vector construction, codon optimization, regulatory components, protein localization and glycosylation (Amack and Antunes, 2020; Jin et al., 2022; Mirzaee et al., 2022; Moon et al., 2022; Zhao et al., 2023).

Systems Engineering in biology can be defined as a holistic approach that analyzes, models, alters, optimizes, and regulates the complex processes of biological systems resulting in desired functions. Artificial Intelligence (AI) refers to the development of machines and systems that use algorithms and statistical models to analyze data, identify patterns and can perform/outperform tasks that demand human intelligence in learning, reasoning, planning, communicating, and problem-solving (Russell, 2010). Machine Learning (ML) is a subset of AI that enables the systems to learn by providing abundant training datasets and is classified into supervised, unsupervised and semi-supervised learning algorithms. Supervised algorithms are the most used of the three since they are developed using labelled datasets from databases with minimum data redundancy, feature extraction, analysis & selection of main traits, prediction methods, and performance evaluation. They provide an excellent prospect for biologists in identifying patterns of gene expression and relevant features, thereby governing the identification through deep understanding of different combinations of the responsible factors (Singh et al., 2016; Silva et al., 2019). Deep Learning (DL) is a network-based supervised learning method with multiple layers of simple modules pooled and arrayed for learning, computing, and mapping a big dataset through each layer. It takes advantage over other AI-based ML algorithms in exploring complex structures of high-dimensional data built from the simplest layers (Lecun et al., 2015). Industry 4.0 revolutionizes traditional practices of manufacturing in industrial settings with the integration of digital technologies, automation, and data exchange, which concourses physical and digital systems leading to increased efficiency, productivity and innovation. Intervention of automation, cyber-physical systems, internet of things (IoT) and big data analytics would prove to be efficient and robust in plant-based biologics production (Dubey et al., 2018; Chen et al., 2020).

**Figure 1:**
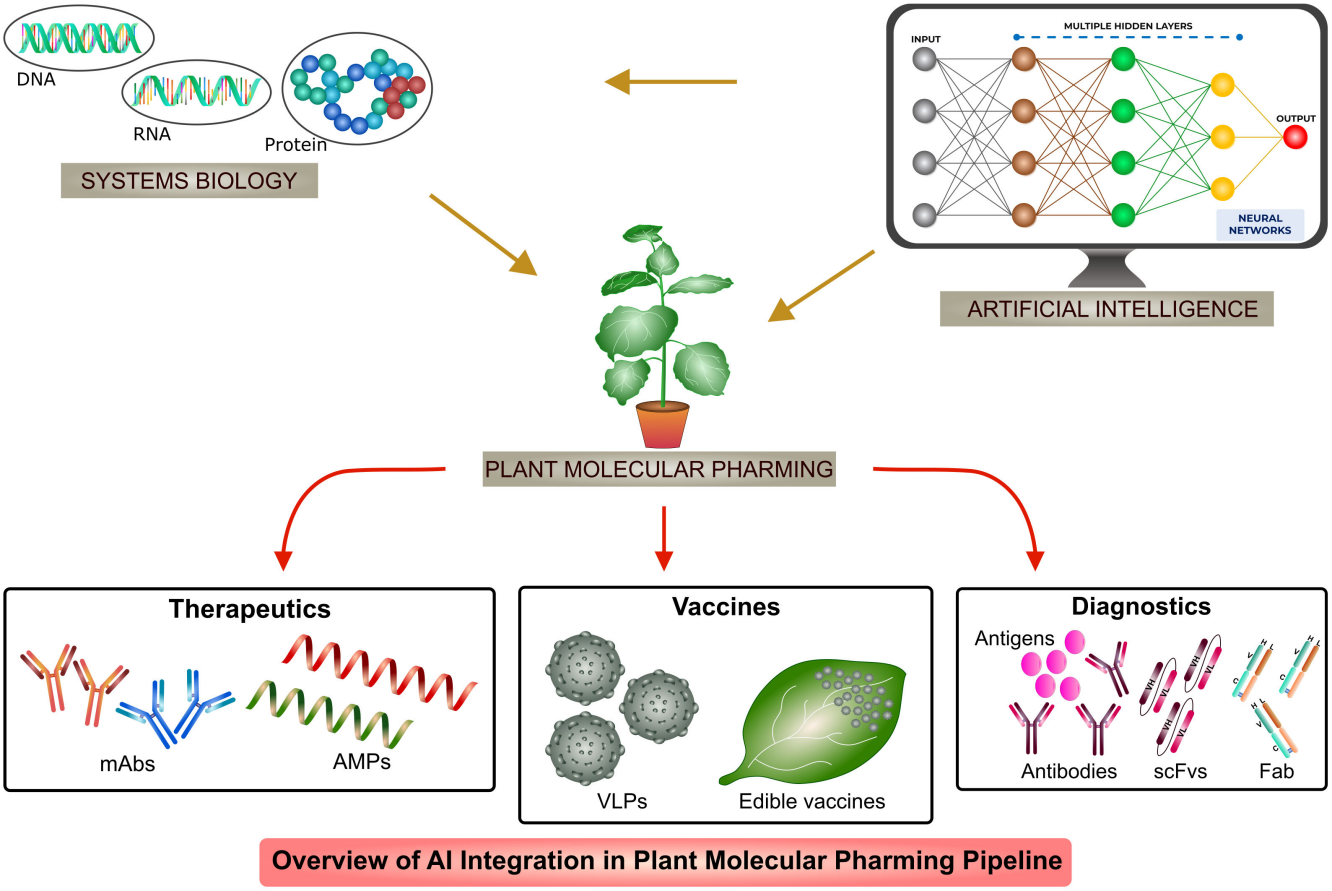
Overview of AI integration in plant molecular pharming pipeline.

AI has been used in recombinant biologics production in host systems such as mammalian cells (CHO and HEK293), yeast (*Saccharomyces cerevisiae* and *Pichia pastoris*) and bacterial (*Escherichia coli* and *Bacillus subtilis*) systems (Van Brempt et al., 2020; Smiatek et al., 2021; Feng et al., 2022a; Li et al., 2022a; Packiam et al., 2022). Application of AI or ML algorithms include protein engineering, protein-protein interaction, stability, localization, solubility, functional motif prediction and catalytic activity which increases the production and functionality of recombinant proteins (Han et al., 2019; Jiang et al., 2021; Feng et al., 2022a; LaFleur et al., 2022; Masson et al., 2022; Kalemati et al., 2023). Till date, AI finds very least or no intervention in plant molecular pharming. In this review, we discuss about the systems biology concepts with the introduction of AI, as shown in **Figure 1**, in different aspects of recombinant biologics production to increase the stability, functionality and applications of AI-based ML algorithms in engineering systems to overcome the challenges and to enhance the production of next generation plant-based biologics.

## 2 Advantages of plant expression system

The market size of plant-based biologics was valued at $116.1 million during the year 2021, and with the compound annual growth rate (CAGR) at 4.8%, it is being estimated to reach $182.9 million by the year 2031. Few of the major plant-based production firms include Leaf Expression Systems, Zea Biosciences, Plant Biotechnology Inc., InVitria, Mapp Biopharmaceutical and PlantForm (Allied Market Research, 2023). Very few plant-based recombinant therapeutics have been commercialized following development and many are under clinical trials (He et al., 2021; Lobato Gómez et al., 2021). Elelyso, taliglucerase alfa, produced in carrot cell culture by ProtalixBio Therapeutics was approved by FDA in 2012 to treat Gaucher disease and has been commercialized (Mor, 2015). ZMapp – an antibody cocktail produced in *N. benthamiana* by Leaf Biopharmaceutical (commercialization arm of Mapp Biopharmaceutical) was used to treat Ebola outbreak under emergency use authorization during 2014 in Africa (Qureshi, 2016). Recombinant growth factors were produced in the endosperm of barley grain by ORF Genetics and have been commercialized as skincare products (ORF Genetics, 2023). Covifenz, a plant-based SARS CoV2 VLP vaccine against COVID19, developed by Medicago was authorized by Health Canada during 2022 (Hager et al., 2022).

Protein-based pharmaceutical products are growing rapidly in recent years and most of them are produced in mammalian and microbial expression systems. Now-a-days, plant systems have emerged as an alternative platform for large scale production of recombinant proteins as they necessitate no capital-intensive infrastructure, bioreactors, or expensive culture media, but may be quickly scaled in low-cost greenhouses using simple reagents (Chen and Davis, 2016). When compared with prokaryotic and other host systems, plants offer an alternative bioreactor system for recombinant expression due to their glycan profile and cost-effective management system (Schillberg et al., 2019). Apart from the advantages mentioned above, plant systems are human pathogen free, sterile conditions are not required during production and scalable due to open-field cultivation (Buyel, 2019). For all these reasons plant expression system has been established as a prominent bioreactor for the production of therapeutic proteins such as vaccines, therapeutic proteins and growth hormones (Limkul et al., 2016; Moon et al., 2022).

Each expression host has its advantages and limitations. For instance, mammalian cell systems are capable of inherently producing recombinant biologics in humanized form, but it is difficult to maintain cell lines free from human pathogens and contaminants (Sethi et al., 2021). Plant system has many advantages over other systems including rapid (production of recombinant protein starts at day 2-3 post infiltration), cost-effective (produced at a cost of $0.27 for 3 mg dose of recombinant AMP), scale-up (increasing the plant biomass as required and thereby protein yield), purity (up to 99%), safety (production without any contaminant interference and functionally safe in humans) and post-translational modifications (*N*-glycosylation in engineered tobacco plants, which prokaryotic host system lacks). These advantages can be briefed with an example each using *N. benthamiana* transient expression host system. SARS-CoV2 RBD (Receptor binding domain) Fc fusion vaccine candidate was expressed in *N. benthamiana* and was extracted 4 days post infiltration which gave an yield of 25 µg/g FW (Siriwattananon et al., 2021). Alam et al. (2018) were able to produce antiviral compound Griffithsin at 99% purity from tobacco plant. Two mAb isotypes, 4H2 IgG and 4H2 IgM antibodies against *Coccidioides* CTS1 (Valley Fever) antigen were expressed in *N. benthamiana* plants showing homogenous N-glycosylation profile with a dominant GnGn/GnM structure, highly similar to mammals. Techno-economic analysis by McNulty et al. (2020) of *N. benthamiana*-based recombinant protein production reveals that the plant can produce up to 4 g of protein per kg FW (g/kg FW) with the yield up to 300 kg of recombinant protein per year through transient expression.

## 3 Systems engineering approaches to produce recombinant biopharmaceuticals in plants

Plant-based biologics have emerged as a promising alternative for therapeutics production due to their low-cost and scalable nature. This is critical for meeting the demand for immunizations during pandemics. Production of recombinant therapeutics in plants can be achieved by either stable or transient expression. Stable expression systems are developed by nuclear transformation or chloroplast transformation through *Agrobacterium*-mediated or biolistic gene transfer (Gelvin, 2003; Tien et al., 2019; Bolaños-Martínez et al., 2020; Heenatigala et al., 2020; Kumar and Ling, 2021). Meanwhile, transient expression systems are developed by plant virus-based vectors or agroinfiltration. Stable expression systems possess advantages including scale-up, low storage costs, glycosylation patterns and reduced cross contamination of animal-borne agents; Transient expression systems are known for their rapid, cost-effective, increased protein accumulation and commercialization potential (Moon et al., 2019). Transient expression of recombinant biopharmaceuticals in plant system is the most preferred mode of production since the system accumulates large quantities of proteins quickly. Different immunogens and therapeutic agents have been produced through transient expression in leaves by agroinfiltration (Iyappan et al., 2018; Page et al., 2019; Rattanapisit et al., 2020).

Proteins reach functional state by proper folding, disulphide bond formation, subunit assembly and post-translational modifications. Prokaryotic host systems pose limitations such as lack of post-translational modifications (glycosylation and sialylation), signal peptide cleavage and pro-peptide processing (Gomord and Faye, 2004). Glycosylation is the most prevalent and diverse type of post-translational modification of proteins shared by all eukaryotic cells. A complex metabolic network and many glycosylation pathways are used during the enzymatic glycosylation of proteins to produce a wide variety of proteoforms (Schjoldager et al., 2020). For instance in humans, N-acetylglucosaminyl transferases IV and V present in Golgi functions in galactosylation, branch elongation and sialic acid capping, which is not found in plants (Strasser, 2022; Strasser, 2023). In order to produce therapeutic proteins of interest in plant with desired glycosylation pattern, β-1,4 galactosyl transferase co-expression and sub-cellular localization to Golgi is preferred (Navarre et al., 2017; Strasser, 2022). Recombinant glycoproteins produced in plants have residues of α1,3-fucose and β1,2-xylose linked to the same core N-glycan. These two sugar residues could be immunogenic since they are absent in human glycoproteins (Margolin et al., 2020a). In Arabidopsis, tobacco, and rice, multiplex CRISPR-Cas9 technology was used to knock out two glycosyl transferases, β1,2-xylosyltransferase and α1,3-fucosyltransferase, in order to humanize glycosylation patterns in plants and produced biopharmaceuticals. The results demonstrate that complete suppression of these two sugar residues was reported in Arabidopsis and tobacco, while the presence of Lewis structure in rice shows that the glycosylation pattern differs between dicots like Arabidopsis and tobacco and monocots like rice (Jansing et al., 2019; Jung et al., 2021). Many therapeutic proteins that are glycosylated need to be sialylated ultimately to fully activate their biological functions, however plants are not capable of N-glycan sialylation, in contrast to mammals. The ability to perform N-glycan sialylation is much sought after in the plant-based biopharmaceutical industry since sialic acids are a frequent terminal alteration on human N-glycans. Plants can be engineered across α2,6-sialylation or α2,3-sialylation pathways that showed active IgG with anti-inflammatory properties and increased pharmacokinetic activity of therapeutics produced in plants (Strasser, 2023). N-glycan sialylation is highly desirable due to its function in extended half-life, stability, solubility, and receptor binding (Bohlender et al., 2020; Chia et al., 2023). A whole mammalian biosynthetic pathway, including the coordinated expression of the genes for (i) biosynthesis, (ii) activation, (iii) transport, and (iv) transfer of Neu5Ac to terminal galactose, has been introduced into *N. benthamiana* in order to achieve *in planta* protein sialylation (Izadi et al., 2023).

Recombinant biologics expressed in plants are designed as fusion proteins to contain an N-terminal or C-terminal tag (His, FLAG, HA, CBM3 etc.) for easy purification and analysis. Immobilized metal-ion affinity chromatography is widely used for purification of hexahistidine tagged proteins (Vafaee and Alizadeh, 2018; Islam et al., 2019; Hanittinan et al., 2020; Islam et al., 2020; Marques et al., 2020; Soni et al., 2022). Other techniques such as one-step cation-exchange chromatography, Protein G-/A-based affinity chromatography, diafiltration (antibody purification) and polyelectrolyte precipitation (removal of plant proteins), hydrophobic interaction chromatography (HIC) followed by hydrophobic charge induction chromatography (HCIC) are employed in recombinant plant protein purification (Fulton et al., 2015; Park et al., 2015; Shi et al., 2019; Miura et al., 2020; Lim et al., 2022; Grandits et al., 2023).

## 4 AI-based ML algorithms in recombinant protein production

**Figure 2:**
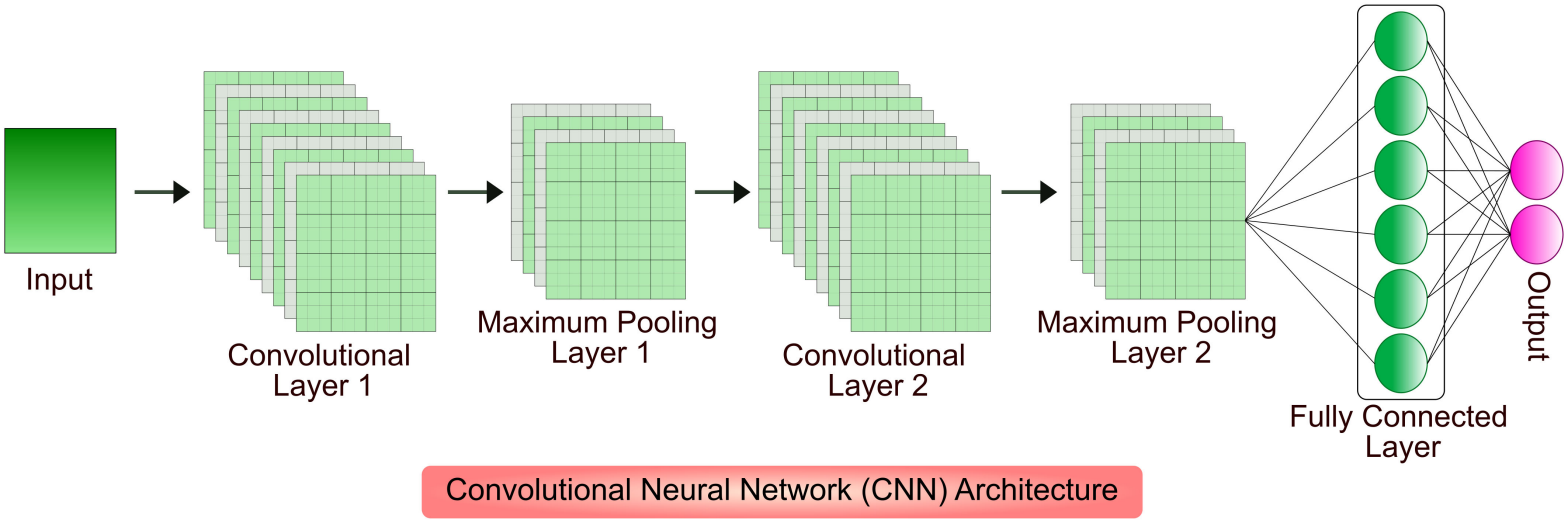
An illustration of input characteristics recognition using CNN. The input spatial features pass through multiple convolutional and pooling layers; processed data is received at a fully connected layer. The convolutional layer applies filters to extract features from input, pooling layer downsamples the features in order to reduce computation and fully connected layer makes the final prediction to result the output.

Gene designing and genetic engineering are key tools in molecular pharming, which enable the expression of protein of interest in host system, and development of genetically modified organisms with desirable traits. The design of gene and its expression cassette is the first step in getting desired protein in the plant system (Rozov and Deineko, 2019). Proper designing plays a major role in the production of biologics that includes selection of host system, codon optimization, regulatory components associated with foreign gene, host engineering, mode of expression, and purification of biopharmaceuticals (Webster et al., 2017; Peyret et al., 2019; Belcher et al., 2020; Sainsbury, 2020; Hassan et al., 2021; Vazquez-Vilar et al., 2023). AI-based ML algorithms are proven choice for cost-cutting and efficient designing of product manufacturing in different host systems. Few of the competent network models were built on Convolutional Neural Networks (CNNs), a DL architecture inspired from connectivity patterns of animal visual cortex to identify, locate and differentiate objects in any image (Barré et al., 2017). Different AI-based ML and DL algorithms have been developed to increase the recombinant biopharmaceutical production in the hosts by detecting, analyzing and optimizing the conditions such as screening and candidate selection, vector construction, codon optimization, protein modelling and design, growth condition optimization and protein solubilization and purification. A model architecture of CNN is shown in **Figure 2**.

### 4.1 AI in codon optimization

Introduction of native genes into alternate host system causes incompatibility in codon usage bias, sequence repeats, % of GC, negative cis-regulatory elements and Shine-Dalgarno sequence (Tuan-Anh et al., 2017; Constant et al., 2023; Jain et al., 2023). Codon bias affects the expression of transgene in the host plant which result in stopping at disfavored codons, truncation, misincorporation or frameshift. Site directed mutagenesis can resolve these problems by introducing silent mutations in coding region of the transgene and help the host species read transgene codon without any hindrance (Ma et al., 2003). Heterologous expression of recombinant proteins in different hosts needs optimization of coding sequences with synonymous codons as the host systems tend to remove heterologous proteins through proteolysis. Further, codon optimization renders the recombinant protein with structural and functional conformation at increased levels of expression in different host systems (Al-Hawash et al., 2017; Argentinian AntiCovid Consortium, 2020; Ding et al., 2022). The codon optimization percentage is proportional to the level of recombinant transgene expression. The amount of expression of the four variants of the *bar* gene with varying percentages of optimized codons was examined using experimental and *in silico* methods, and it was found that genes with 50–70% of optimized codons were expressed effectively in *N. tabacum* (Agarwal et al., 2019). Beta-defensin from chicken called chicken β Gallinacin-3 has demonstrated broad-spectrum antibacterial action against plant infections. Using DNAWORKS3.0 and the Genscript Rare Codon Analysis Tool, chicken β Gallinacin-3 gene sequences were codon optimized and tested. The results demonstrated constitutive expression in *Medicago sativa* and improved antibacterial activity against *E. coli*, *S. aureus*, and *Salmonella typhi* (Jin et al., 2022). Despite species difference, the codon optimizer program improved translation efficiency in tobacco and lettuce by using codon usage hierarchy of the *psbA* gene (Kwon et al., 2016). Adiponectin, an adipokine and a cell signaling protein, is produced as a secretory protein in *Withania somnifera* hairy root culture. Codon usage data, base composition and codon adaptive index (CAI) of *W. somnifera* were analyzed; the human adiponectin gene sequence was optimized and expressed as secretory product. Optimization of codons increased the expression levels of protein secretion (Dehdashti et al., 2020). The synthesis and expression of therapeutic proteins depend heavily on codon optimization. Effective methods are required to efficiently optimize codons for the generation of recombinant proteins in plants (Webster et al., 2017). Codon usage bias was utilized to optimize nucleotide sequences for host-specific expression in many systems including *E. coli*, Chinese Hamster Ovary (CHO) cells, HEK293, etc (Al-Hawash et al., 2017; Shayesteh et al., 2020; Lu et al., 2021). Till date, no AI tool has been designed to optimize codons for increasing the plant-based recombinant biologics production. The challenges posed by conventional methods include a vast possibility of codon combinations, irrational effects following transcription and translation, protein misfolding and loss of function (Constant et al., 2023).

**Figure 3:**
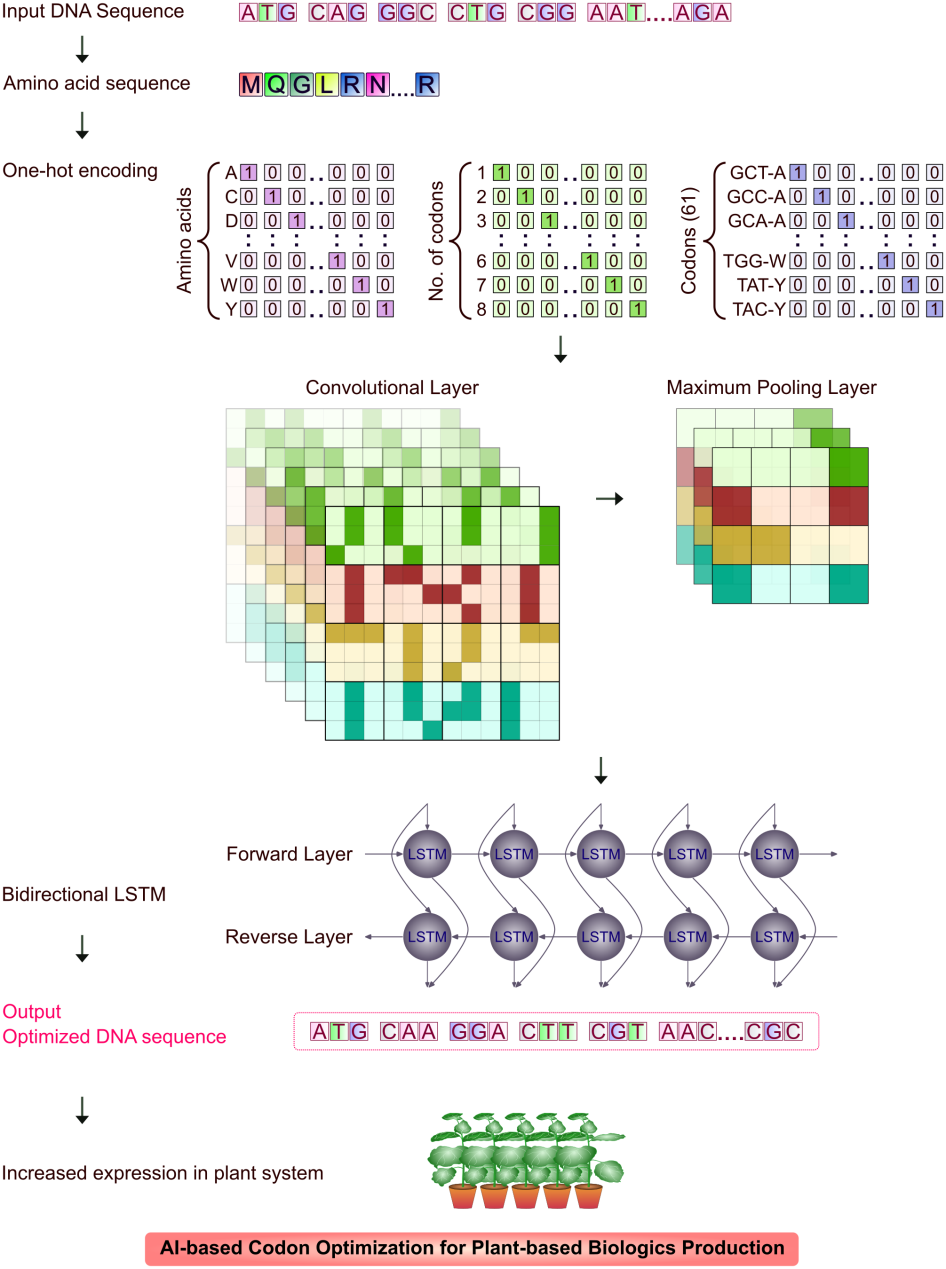
An overview of CNN and BiLSTM in codon optimization for plant expression system. Input DNA sequence is translated to amino acid sequence and reverse translated to DNA sequence. The sequence is further tested using CNN for codon optimization metrics prediction for plant expression system and BiLSTM tests with synonymous codons for optimal codon usage for high expression in plant system.

Neural network (NN) models identify unexplored patterns in the native DNA sequences from the training set, predicts the most valid coding sequences using the test set and optimize DNA sequence for translation. The NN-optimization is found to be more efficient than conventional methods resulting in significantly higher yields of recombinant biologics (Goulet et al., 2023). Many sequence-based ML algorithms using deep neural networks (DNN) extract features from input codon data, predict and evaluate sequence data. Two major parameters that play a crucial role in codon optimization are 1) codon adaptation index (CAI) and 2) tRNA adaptation index (tAI). CAI is the frequency of codon usage in an organism’s coding DNA sequence (CDS) and tAI is the measure of intracellular tRNA to translate into proteins and individual codon-anticodon pairing efficiency (Sabi et al., 2017; Tuan-Anh et al., 2017; Fu et al., 2020; Constant et al., 2023; Goulet et al., 2023). A Recurrent Neural Network (RNN) model trained sequence was tested for its efficiency by transient transfection of unoptimized and optimized sequences in CHO (ExpiCHO) cells. The titres of model protein, human programmed death ligand 1 (PD-L1) extracellular domain, were quantitated nine days after transfection. The RNN-optimized sequence was expressed largely (179.5 ± 12.4 μg/mL) than the native sequence (104.5 ± 5.7 μg/mL). The RNN model was used in optimization of mAb and stable integration of mAb CDS in CHO-K1-derived cells. The RNN-optimization of CDS yielded 2030 μg/mL and the unoptimized sequence resulted in an yield of 960 μg/mL (Goulet et al., 2023). Influence of AI in bacterial expression system is more than any other eukaryotic systems and so codon optimization was widely carried out through ML-based models. Tuan-Anh et al. (2017) used neural network with CAI and GC content for optimizing codons expressing prochymosin, the chymosin-precursor in *E. coli* system. Codon optimization could preferably not just used for increasing heterologous recombinant expression, but also for increasing the protein solubility. MPEPE, a newly developed protein solubility prediction DNN model was built using convolution layers, pooling layers and long-short term memory (LSTM) layers. The architecture was built as embedded matrix, through ‘one-hot encoding’ technique using integers ‘1’ and ‘0’, to include synonymous codons of individual amino acids. Point mutation in sites was scrutinized through evolutionary analysis without interfering the protein function. The target nucleotides for expression studies were used as inputs in MPEPE for virtual screening and recombinant proteins were expressed in *E. coli* BL21 (DE3) cells with an increased level of soluble protein expression (Ding et al., 2022). Bidirectional LSTM Conditional Random Field (BiLSTM-CRF) model is a codon optimization model built for *E. coli* by H. Fu et al. (2020). The model converts codon optimization to sequence annotation and trains the data of *E. coli* gene set through word-embedding vector. The multivalent *Plasmodium falciparum* vaccine antigen FALVAC-1 and PTP4A3, a prognostic cancer biomarker optimized by BiLSTM-CRF were expressed in *E. coli* BL21 (DE3). The model efficiently optimized the low-expression candidate to higher expression levels, which proved the robustness of the model and the high expression candidate PTP4A3 was expressed in similar levels which proved the stability of algorithm. Jain et al. (2023) designed ICOR (Improving Codon Optimization with RNNs), a DL tool, built on BiLSTM architecture through ‘one-hot encoding’ method, with a large non-redundant dataset of *E. coli* genomes and upon correlation comparison with the mRNA expression in real-time based on a work by dos Reis et al. (2003), the improvement in expression observed was about 236%. The multilayer network model may be trained for other host systems including model plants (such as *N. benthamiana* or *N. tabacum*) as shown in **Figure 3** with complete omics dataset through transfer learning approach to increase the yield. CO-BERTa, a deep contextual language model was trained with GFP (Green Fluorescent Protein) and anti-HER2 VHH CDSs on *Enterobacterales* dataset for functional protein measurement. The mCherry reporter protein which showed 28.7% pairwise identity to GFP and anti-SARS-CoV2 VHH which showed 73.7% pairwise identity to anti-HER2 VHH was chosen to test the model. These proteins differ in their length but share similar structural features, a major feature being *β*-barrel. ACE (Activity-specific Cell Enrichment) measurement of CO-BERTa codon optimized proteins in SoluPro™ *E. coli* B strain showed highest expression levels than commercial algorithms (except Genewiz, p<0.05) (Constant et al., 2023). Further, genome analysis and codon usage patterns of plant host systems through artificial neural networks (ANNs) could significantly increase the expression of recombinant biologics (Doyle et al., 2016).

Quantum computers can be used to optimize codons for high expression of proteins. Quantum Annealing (QA) algorithm uses quantum computers to give high-dimensional combinatorial optimization of codons using Binary Quadratic Model (BQM) built on ‘one-hot encoding’ technique. mRNA codons of peptide fragments and full length proteins of SARS-CoV2 spike glycoprotein were optimized using Quantum Approximate Optimization Algorithm (QAOA) (Fox et al., 2021).

Currently, there are no ML-based algorithms available for codon optimization of recombinant proteins to express in plants. The algorithms available for other host systems could be adapted, remodelled and designed for plant-based expression hosts since many of the model plants’ genome is available publicly.

### 4.2 AI in protein modelling and design

The recombinant proteins expressed in different systems are influenced majorly by factors including structure, solubility, catalytic activity, protein folding and stability. Vector and gene of interest is designed to overcome the challenges of recombinant protein expression. The components of protein modelling include host and expression vector selection, promoter, selectable marker, fusion tags. ML based algorithms enhance the expression and overcome the challenges in expression of recombinant biologics in multiple expression systems. These algorithms analyses and tests (either nucleotides – CDS/RNA-seq or amino acids) sequences and provides with the fitness of protein variants (Wittmann et al., 2021). Few ML models utilize structure along with sequences of amino acids for modelling of proteins. The RNNs and other neural network models are powerful than other ML models since these could learn from raw data directly without any sequence alignment and heuristic scoring (Deep RNN for Protein Function Prediction from Sequence). While molecular dynamics simulations for an antibody through supercomputers require hours and even days, neural networks such as CNN models take only seconds to get the work done in personal computers (Lai, 2022). Regulatory elements are one of the key components of recombinant protein production and synthetic promoters have been designed using ML models to increase the transcription efficiency. Highly functional Synthetic Promoters with Enhanced Cell-State Specificity (SPECS) were identified from a library of 6107 promoters using multiple ML regression algorithms, from which a generalized linear model with elastic net regularization (GLMNET) was chosen as the efficient model to predict highly active promoters. The spatiotemporal activity of each promoter was analyzed by expression of fluorescent protein in HEK-293T cells (Wu et al., 2019). In the work by Vo ngoc et al. (2020), human PolII core promoter was analyzed to create HARPE (high-throughput analysis of randomized promoter elements). The HARPE training dataset included 200,000 variants of promoter sequences and downstream core promoter region (DPR) models were generated by support vector regression (SVR) algorithm and tested *in vitro* and in HeLa cells. Designing protein includes predicting counterparts, which are involved in structural integrity and stability of proteins (Masson et al., 2022). These include epitope prediction, vaccine designing and remote homology detection, which utilize parts of the protein molecule to increase its activity (Mettu et al., 2016; Moss et al., 2019; Yang et al., 2021b; Koşaloğlu-Yalçın et al., 2022; Routray et al., 2022).

Using DeepLoc, a deep convolutional network Kraus et al. (2017) showed improved performance over traditional approaches in the automated classification of protein subcellular localization in yeast cells. Organelle targeting and sub-cellular localization increases the recombinant therapeutic protein expression in plants to higher levels. Localization of recombinant proteins in cytosol and different plant organelles such as nucleus, chloroplast, mitochondria and endoplasmic reticulum (ER) of plant tissues such as seeds and leaves are useful in increased accumulation and stability of expressed proteins (Vafaee and Alizadeh, 2018; Arcalis et al., 2019; Bidarigh fard et al., 2019; Islam et al., 2019; Shi et al., 2019; Hanittinan et al., 2020; Islam et al., 2020; Li et al., 2022b; Lim et al., 2022). Signal sequences are added to N-terminus or C-terminus of the biologics to increase the yield and a C-terminal ER retention signal is the most widely used strategy to accumulate higher amount of proteins in recombinant expression. Sahu et al. (2021) developed a tool, Plant-mSubP, based on integrated ML approaches with SVM as the model to predict localization of proteins to single and dual organelle targets.

Analysis of the enriched bococizumab yeast cell libraries along with similar library for antibody affinity was done using an ML model, which enabled the identification of rare variants with co-optimized levels of low self-association and high affinity (Makowski et al., 2022). Similarly, mAbs can be screened and optimized for production in specific host systems that could include plants as well (Feng et al., 2022a; Lai, 2022). Proteins such as toxins which are difficult to produce in certain hosts can be expressed easily using deep-learning based CNN algorithms (Pan et al., 2020). A wide range of ML algorithms used in various eukaryotic and prokaryotic systems for modelling different proteins is shown in **Table 1**.

**Table 1:** AI in protein modelling and design.

Component	Name of the program	Type of ML algorithm	Architecture	Function/Parameter	Model system/training dataset	References
mRNA	APARENT (APA REgressionNeT)	CNN	One-hot encoded matrix system with two convolutional layers	**• **mRNA isoform prediction and polyadenylation within +10 to +35 nt downstream of 6-base central sequence element (CSE) **• **cleavage site prediction across polyA signal	HEK293	Bogard et al. (2019)
	6-mer Logistic Regression Baseline	Linear logistic regression	One-hot encoded matrix system with 6-mer counts	**• **mRNA isoform prediction and polyadenylation **• **cleavage site prediction		
mRNA, gene enhancers and protein	DEN (Deep Exploration Network)	Deep Convolutional Generative Adversarial Networks (DC-GANs)	One-hot encoded matrix Latent Seed Sequence Tensor	**• **polyadenylation signals conformed to mRNA isoforms and 3’ cleavage sites **• **differential splicing **• **maximum transcriptional activation of gene enhancers **• **functional variants of GFP (Green Fluorescent Protein)	HEK293 HeLa MCF7 CHO	Linder et al. (2020)
	APARENT	CNN				
	-	GP regression				
	APA VAE (Variational Autoencoder)	Residual Neural Network (ResNet)				
	KL-bounded DEN	CNN				
Gene interaction and expression	scCapsNet	DNN	Capsule Neural Network	Discovery of gene interactions; closely related in function but presenting differential gene expression pattern in single cell types (based on transcriptome analysis)	scRNA-seq dataset including mouse retinal bipolar (mRBC) cells and human peripheral blood mononuclear cells (hPBMC)	Wang et al. (2020)
Transcription factor	Independent Component Analysis (ICA)	Unsupervised ML	-	Gene expression and transcriptional regulation in *E. coli* through transcriptome analysis	*E. coli* K12 RNA-seq expression profiles	Sastry et al. (2019)
Transcription factor binding	FactorNet	Convolutional RNN	One hot encoded 4-row bit matrix, LSTM	Transcription Factor (TF) cell type specific binding site prediction. (Eg.TF E2F1 binding to GM12878 and HeLa-S3)	DNase-seq, ChIp-seq and RNA-seq data of chromosomes X and 1-22 from ENCODE-DREAM challenge	Quang and Xie (2019)
Promoters	Hybrid biophysical-ML approach	Ridge regression model	-	**• **Synthetic promoter designing **• **Identification of -35 and -10 motifs and optimal spacer length	*E. coli*	LaFleur et al. (2022)
Synthetic promoter	DL model	Deep CNN	Transformer model with BiLSTM	Design regulatory sequences including orthologous promoters	RNA-seq data from *S. cerevisiae* and 10 other Ascomycota species	Vaishnav et al. (2022)
Protein	DeepRHD	DNN	CNN based bidirectional GRU (Gated Recurrent Units)	Remote homology prediction of protein sequences using physico chemical properties and evolutionary information	SCOP1.67 dataset	Routray et al. (2022)
Protein	ProtT5	pLM (protein language models) Logistic Regression	Attention based deep dilated residual networks consisting of convolution layers (ResNet CNN)	Protein (transmembrane beta barrel proteins – OmpX and variants) structure prediction from sequences	High resolution protein 3D structure dataset from ProteinNet12	Weissenow et al. (2022)
Protein	ML model	Linear regression models including glmnet, partial least squares, averaged neural network, SVM with radial basis function kernel, stochastic gradient boosting, boosted generalized linear model, random forest, cubist and naïve Bayes models	Caret package in R	Factors influencing recombinant protein stability including Molecular weight, cysteine residues and N-linked glycosylation	CHO cells expressing human secretome	Masson et al. (2022)
Protein	ASPIRER	DL model	XGBoost and N-terminal sequence-based CNN	Prediction of Non-classical secreted proteins (NCSPs)	Gram positive bacteria NCSPs dataset from UniProt	Wang et al. (2022)
Protein	eUniRep	DL NN	UniRep multiplicative LSTM	Protein, avGFP and TEM-1 β-lactamase, engineering (Low-*N* engineering) using small number of functional variants	*E. coli* DH5α	Biswas et al. (2021)
Protein	UniRep	SVM	RNN	Prediction of recombinant gene expression and protein solubility	*B. subtilis*	Martiny et al. (2021)
		LR				
		Random Forest (RF)				
		ANN				
Protein	ECNet	RNN	BiLSTM, Transformer architecture with TAPE integration	Protein fitness prediction based on evolutionary context, engineered TEM-1 β-lactamase variants showing enhanced ampicillin resistance	*E. coli* DH5α Diverse large-scale deep mutational scanning (DMS) datasets and random mutagenesis datasets	Luo et al. (2021)
Protein	EPSOL	Keras based DL model	Multidimensional Embedding, multi-convolutional-pooling module and a Multi-layer Perceptron (MLP)	Protein solubility prediction	Heterologous expressed *E. coli* soluble and insoluble protein dataset compiled by Smialowski et al. (2012)	Wu and Yu (2021)
Protein	DEEPred	Multi-layered perceptrons (MLPs)	Feed-forward multitask DNN	Sequence/Gene Ontology (GO) based functional definition prediction of proteins	*Pseudomonas aeruginosa* strain reference genome and UniProtKB/Swiss-Prot dataset	Sureyya Rifaioglu et al. (2019)
Protein	ML models	GANs	Generator Neural Network and Discriminator Neural Network	Prediction of Protein solubility	eSol database dataset	Han et al. (2019)
		Logistic regression				
		Decision Tree				
		SVM				
		Naïve Bayes				
		Cforest				
		XGboost				
		ANNs				
Protein	DeepSol	DL model	CNN, non-linear high-dimensional k-mer vector spaces, deep feed-forward neural network (FFNN)	Protein solubility prediction	Heterologous expressed *E. coli* soluble and insoluble protein dataset compiled by Smialowski et al. (2012)	Khurana et al. (2018)
Protein	ML	RNN	BiLSTM, One-hot encoded matrix	Identification and function prediction of protein homologs including iron sequestering proteins, cytochrome P450, serine and cysteine proteases and G-Protein coupled receptors, detection through fluorescence (GFP)	*E. coli*	Liu (2017)
Protein	SPIDER2	Deep learning neural network	Stacked sparse autoencoder	Protein secondary structure, solvent accessible surface area, main chain torsion angle prediction	Non-redundant high resolution protein structures dataset	Yang et al. (2017)
Amyloidogenic proteins	AbsoluRATE	SVM	Sequence-based regression	Aggregation kinetics prediction of amyloidogenic proteins	CPAD 2.0 database dataset	Rawat et al. (2021)
Antibody	DeepAb	Deep residual convolutional network (Deep RCN) with Rosetta-based protocol	RNN, BiLSTM, LSTM	Antibody Fv structure prediction from sequence	Observed Antibody Space (OAS) database, SAbDab database	Ruffolo et al. (2022)
Antibody	DeepH3	Deep residual network	One dimensional and two dimensional convolutions	Prediction of *de novo* CDR H3 loop structures	Rosetta and SAbDab dataset	Ruffolo et al. (2020)
mAbs	solPredict	ESM1b-based Multilayer perceptron (MLP2Layer) transfer learning model	Pretrained protein language model EMS1b embedding	**• **Rapid, large-scale high throughput screening of mAb sequences (IgG1, IgG2 and IgG4) and quantitative solubility prediction eliminating precipitation in Histidine pH 6.0 (H6) buffer system **• **Eliminates the need for 3D modelling	HEK293/CHO	Feng et al. (2022a)
mAbs/IgG1	DeepSCM	Scikit-learn	CNN architecture	Molecular dynamics simulation to screen high concentration antibody viscosity prediction	SAbDab and AbYsis database dataset	Lai (2022)
	Keras v2.7.0	-				
Multiepitope vaccine	DeepVacPred	DNN-V	Multi-layer CNN and a 4-layer linear neural network	Designing vaccine subunit containing both T- and B-cell epitopes of Spike glycoprotein against SARS-CoV2	*E. coli* K12	Yang et al. (2021b)
T-cell Epitope	Antigen eXpression based Epitope Likelihood-Function (AXEL-F)/NetMHCpan 4.1 combination	-	Neural networks	**• **Expression of source antigen; T cell epitope prediction and peptide presentation to MHC Class I molecule **• **SARS-CoV2 epitope prediction	IEDB HLA class I ligands dataset; RNA-Seq data of HeLa cells; SARS-CoV2 expression dataset from Finkel et al. (2021)	Koşaloğlu-Yalçın et al. (2022)
T-cell Epitope	-	Epitope likelihood	Aggregate z-score, structure-based processing likelihood	*P. aeruginosa* endotoxin domain III (PE-III) epitope prediction	*P. aeruginosa*	Moss et al. (2019)
T-cell Epitope	-	Epitope likelihood	Aggregate z-score	CD4+ T-cell epitope prediction in bacterial and viral antigens without genotype information through antigen processing constraint modelling	Sequence data from different studies in C57BL/6 mice, HLA-DR4-transgenic mice and humans	Mettu et al. (2016)
Protein localization	MULocDeep	Bayesian optimization & Attention visualization	LSTM	Protein localization in organelles such as nucleus, mitochondria, plastid and thylakoid and extracellular matrix	Mitochondrial proteome data of *A. thaliana* cell cultures, *Solanum tuberosum* tubers, *Vicia faba* roots	Jiang et al. (2021)
Protein localization	Plant-mSubP	SVM	OvR (One-vs.-Rest)	Single- and dual- organelle targeting/subcellular localization of proteins in plants	Plant protein sequence dataset from Uniprot Database	Sahu et al. (2021)
Cytokines and peptides	ProtConv	Transfer learning CNN	LSTM, ResNet and Transformer with TAPE embedding LeNet-5 architecture	Function prediction of proinflammatory cytokines and anticancer peptides	IEDB and CancerPPD database dataset	Sara et al. (2021)
Peptide	FBGAN (Feedback GAN)	GANs	RNN and Feedback loop training architecture	**• **Generation of synthetic AMPs and α-helical peptide coding genes **• **Optimization of secondary structure	Uniprot database dataset	Gupta and Zou (2018)
Peptide-MHC Class I binding	CapsNet-MHC	CNN	Capsule Neural Network	Prediction of interaction between allelic variants of MHC and peptides with rare sequence lengths	IEDB dataset	Kalemati et al., (2023)
Peptide-HLA binding	DeepSeqPanII	Pan-specific DNN with attention mechanism	LSTM	Prediction of Peptide-HLA Class II binding	IEDB datasets BD2013 and BD2016	Liu et al. (2022b)
MHC Class II Antigen Presentation	NNAlign_MAC	ANN	NNAlign_MA ML framework	**• **CD4 T cell epitope prediction **• **MHC class II antigen presentation prediction **• **Prediction of protein-drug immunogenicity	Single allele and Multiple allele dataset & IEDB dataset	Barra et al. (2020)
Signal Peptide	XGBoost	Regression model	-	Increasing the protein translocation rates to ER by optimizing synthetic signal peptide-protein (mAb/ScFv) complex formation	CHO-K1 cells	O’Neill et al. (2023)
Signal peptide	Sequence-to-sequence model	Attention-based neural network	Transformer model	Signal peptide prediction from Amylase, lipase, protease and xylanase enzymes	*B. subtilis*	Wu et al. (2020)
Signal peptide	SignalP 5.0	DL model	Non-linear PSSMs (position specific scoring matrix), BiLSTM and a conditional random field	Peptide identification (three classes including Sec/SPI, Sec/SPII, Tat/SPI) in prokaryotes	Reference proteomes of *E. coli* K12 and *S. cerevisiae*	Almagro Armenteros et al. (2019)
Toxic motifs	ToxDL	Deep CNN	Bidirectional GRU, one-hot encoded matrix	Toxicity assessment of genetically engineered organisms by highlighting toxic motifs and alteration of toxicity	Toxic/venom protein dataset from Animal Toxin Annotation Project in UniProt	Pan et al. (2020)
	Domain2Vec		Skip-gram model			
NSAID	Ensemble Decision Tree (DT)	Extremely Random Tree (ET)	Multiple base trees with bagging strategy	Non-steroidal anti-inflammatory drug, Oxaprozin, solubility in supercritical CO_2_ fluid	Oxaprozin solubility dataset from Khoshmaram et al. (2021)	Alshehri et al. (2022)
		Random Forest (RF)				
		Gradient Boosting	Sequence of base predictors			

### 4.3 ML models in engineering strains for recombinant protein production

A large repertoire of omics data is obtained from the host system at different levels of replication (genome), transcription (transcriptome), translation (proteome), and regulation (metabolome). These data can be used to engineer host cells to improve recombinant protein yield (Ramzi et al., 2020; Samoudi et al., 2021). ML algorithms can be implemented in understanding the genome-scale metabolic models (GEMs), which encompasses hundreds of metabolic pathways and thousands of metabolic reactions. ML can be a stand-alone or a complementary approach, in learning regulatory levels of complex pathways in plants such as transcriptional, translational and allosteric regulation. These ML algorithms are shown to exhibit more robustness than statistical tools (Radivojević et al., 2020; Zhang et al., 2020; Strain et al., 2023).

Multilayer Perceptron (MLP), an NN model was used to analyse the human RNA-seq data from ARCHS4 database based on secretory index (SI) and extrapolated to engineer CHO cells(Zaragoza, 2022). In order to predict yeast cell growth Culley et al. (2020) proposed ML–based data integration techniques, combining gene expression profiles that rigorously assess and compare with computationally generated metabolic flux. A total of 1,143 *S. cerevisiae* mutants were tested and 27 machine learning methods were analyzed.

ART (Automated Recommendation Tool) and EVOLVE algorithm are ML-based Bayesian ensemble optimization tools used in increasing the production of tryptophan in yeast, *S. cerevisiae*. These ML algorithms were used to design 30 different promoter combinations from the transcriptome dataset, which were used to predict engineered strains to show increased productivity. The engineered strain SP606 was found to possess higher synthesis rate of proxy GFP than other strains designed using ML and library preparation. Also, the engineered yeast strain SP606 was identified to have an increased titre and productivity of tryptophan (Zhang et al., 2020). ART was also trained with concentration dataset of proteins/enzymes involved in heterologous pathway for the production of limonene. New strain design sets of *E. coli* for enhanced production of limonene were provided by ART(Radivojević et al., 2020).

Similarly, supervised learning algorithms have predicted pathway dynamics with the use of multiomics data (proteome and metabolome data) in *E. coli* for enhancing limonene production (Costello and Martin, 2018). In contrast, an unsupervised ML approach termed as HybridFBA, was proposed by Ramos et al. (2022) that combined GEM and metabolic flux balance analysis (FBA) using principle component analysis (PCA) in CHO cells (Strain et al., 2023). Machine Learning Predictions Having Amplified Secretion (MaLPHAS) by Eden Bio Ltd is an ML algorithm that predicted knock out of five genes, out of which Component of Oligomeric Golgi Complex (*cog6*) knockout strain resulted in doubled secretion of recombinant protein in the host *Komagataella phaffii* (*P. pastoris*) compared with the *bgs7 *supersecretor strain (Markova et al., 2022).

DCell is a virtual eukaryotic cell composed of 2,526 subsystems embedded as VNNs (visible neural networks), a deep ANN, in hierarchy. The model was built using the hierarchical architecture of subsystems of *S. cerevisiae*. Being trained on several million genotypes, during simulation, DCell generates patterns of molecular activities based on genotype to phenotype relationship (Ma et al., 2018). DCell can identify gene deletions/knockouts using Gene Ontology (GO), which will result in phenotype change (Ma et al., 2018; Kim et al., 2020).

The ML algorithms and tools can be used to introduce or remove genes from a pathway to direct the increased production of humanized recombinant biologics in plant system. Knock-out approach of removing plant-specific glycans [β(1,2)-Xyl and α(1,3)-Fuc] or knock-in strategy to express human [β(1,4)-Gal]and addition of sialic acid residues in specific host plants result in humanized protein expression. Such mechanisms could be explored and analyzed through ML tools such as ART (Sethi et al., 2021). Also, metabolic flux of host plant systems can be studied to generate stable lines with optimized metabolic pathways for desired post translational modifications of recombinant biologics.

### 4.4 Automation and AI in plant growth monitoring and biomass production

**Figure 4:**
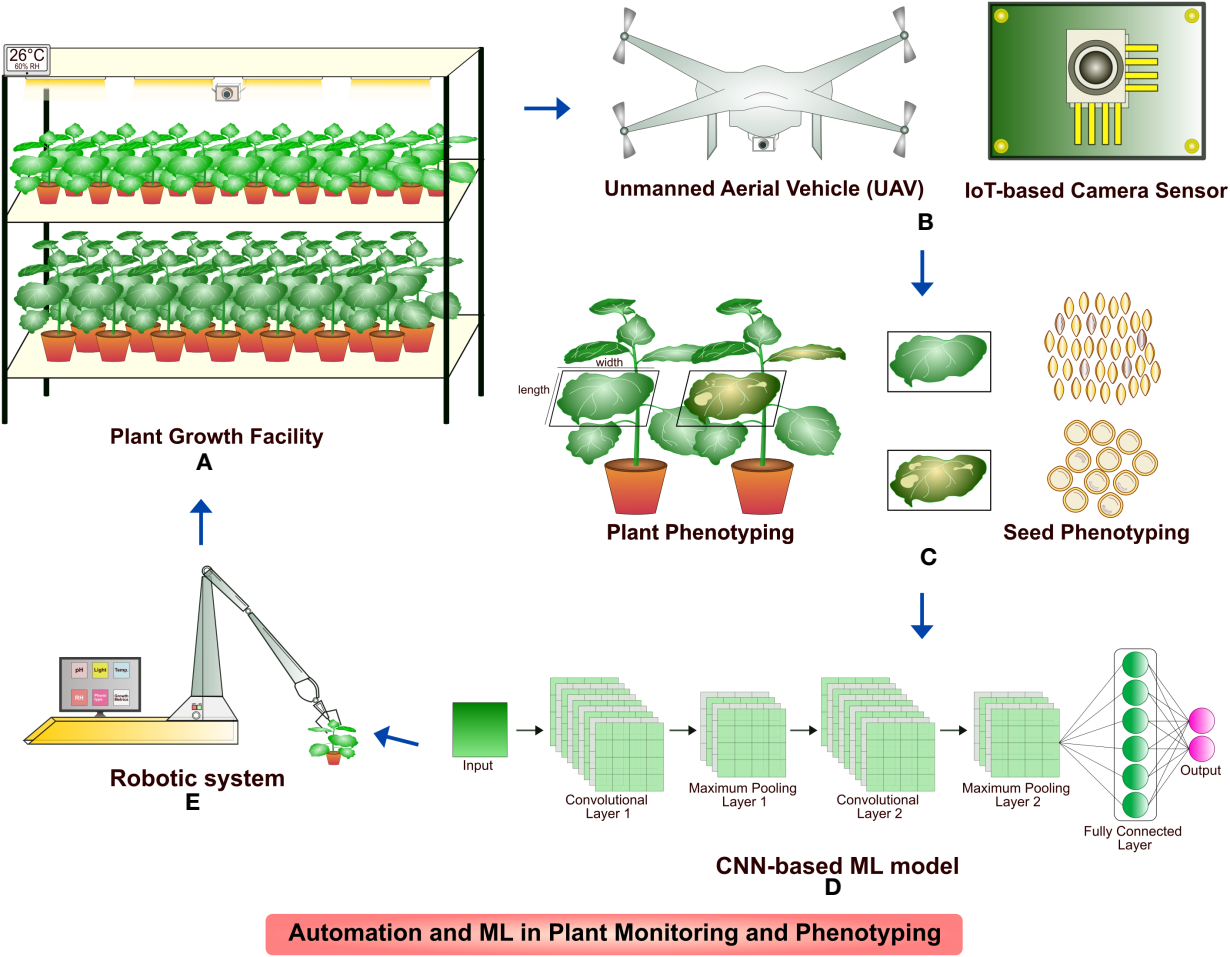
An illustration of plant monitoring and phenotyping with the integration of automation and ML approaches. **(A)** and **(B)** *N. benthamiana* plants grown in containment facility monitored continuously by image sensors; UAV deployed for plant monitoring in greenhouse; **(C)** Image acquisition of plant phenotypes such as height, width, leaf greenness, disease identification, seed count, morphology and segregation; **(D)** Phenotyping of plants by CNN-based ML model; **(E)** Robotic system for optimization and maintenance of environmental requirements for plant growth trained by CNN model.

One of the big attributes of plant molecular pharming for recombinant biologics production, next to host selection and engineering is plant growth and maintenance. Plants are efficient biofactories for the manufacture of recombinant proteins and growth monitoring is a vital aspect when it comes to both laboratory scale and commercial production. Several automation technologies including affordable sensors built on Raspberry Pi, robotics and high-definition cameras work based on image acquisition (Jahnke et al., 2016; Jolles, 2021; Banerjee et al., 2022; Wan et al., 2022). The camera sensors have been deployed to analyze the plant growth patterns, phenotypes such as plant morphology, height, canopy, temperature, leaf biomass, leaf area index, greenness, age and different stresses. Similarly, seed count, shape, size and color, parameters for plant growth such as temperature, photoperiod, grow light color, etc. were studied by robot-assisted systems. A large training dataset of raw images captured in the camera sensors are analyzed through DNN modules and processed for color correction and segmentation for analysis (Jahnke et al., 2016; Ubbens and Stavness, 2017; Tovar et al., 2018; Zheng et al., 2019; Tausen et al., 2020; Bose and Hautop Lund, 2022). The efficient analysis of images are carried out by models based on CNNs that include U-Net, R-CNN and ResNet (Ubbens and Stavness, 2017; Lin et al., 2019; Zheng et al., 2019; Tausen et al., 2020; Bose and Hautop Lund, 2022). The IoT based sensors and programs are not limited to phenotyping the growth and morphology of plants but could detect plant nutrient deficiencies, diseases and soil parameters, thereby reduce the labor intensive maintenance and increase the sustainability (Dhivya et al., 2021; Monteiro et al., 2021; Bose and Hautop Lund, 2022). Plant monitoring and phenotyping using integrated automation and ML approaches is illustrated in **Figure 4**.

With the wider and large-scale biologics production environment, a large number of sensors in plant monitoring are needed and it becomes highly difficult to build the architecture for plant maintenance. Hence remote sensing using unmanned aerial vehicles (UAVs) is used in place at low altitudes to acquire high-resolution multispectral images of plants grown in agricultural field and greenhouses. The UAV high-throughput phenotyping platform, working on support vector machine (SVM) and SVM-derived models, processes the spectral information of optical images for the identification of plant growth, biomass, stress and disease stages (Maimaitijiang et al., 2020; Fu et al., 2021; Yang et al., 2021a; Aslan et al., 2022; Jiang et al., 2022a; Bai et al., 2023a). Several plants used as hosts in production of recombinant biopharmaceuticals such as *Glycine max* (L.) Merr. (soybean), *Triticum aestivum* (wheat), *Hordeum vulgare* (barley), *Oryza sativa* (rice), *Zea mays* (maize), *Arachis hypogaea* L. (peanut), *Arabidopsis thaliana* (Arabidopsis), *Brassica napus* (rapeseed), *Lycopersicon esculentum Mill.* (Tomtato), *Cucumis Linn.* (cucumber), *L. sativa Linn.* (lettuce), *Brassica oleracea linn.* (cabbage), *Raphanus sativus linn.* (turnip), *Apium graveliens Linn.* (celery) and *Spinacia oleracea Linn.* (spinach) and *N. tabacum* (tobacco) can be monitored using the sensors for high product yield (Minervini et al., 2015; Jahnke et al., 2016; Minervini et al., 2017; Ubbens and Stavness, 2017; Zheng et al., 2019; Maimaitijiang et al., 2020; Fu et al., 2021; Sangjan et al., 2021; Sarkar et al., 2021; Yang et al., 2021a; Banerjee et al., 2022; Bai et al., 2023a; Bai et al., 2023b; Sun et al., 2023). A detailed list of automation and AI-based tools used in plant monitoring is listed in **Table 2**. These technologies are not limited to monitoring the mentioned plants but can be extended to all the plant host systems used in expression of recombinant biologics.

**Table 2:** Automation and AI Tools in plant monitoring.

Platform	Automation Technology	Imaging Device	Phenotype/Parameter	Plant Species	References
UAV remote sensing	Multirotor UAV with CNN architecture	XIMEAMQ022MG-CM Camerawith CMOS sensor and 16 mm lens and Sony NEX-7 Camera	Disease severity at 25m altitude	*O. sativa* (rice)	Bai et al. (2023a)
High throughput UAV remote sensing	DJI Phantom 4 Advanced quadcopter	Drone RGB camera	Accurate plant count, location and size determination to distinguish in paddy field at 7m altitude	*O. sativa*(rice)	Bai et al. (2023b)
RiceNet	Deep Learning Network				
Edge-computing based network monitoring	IoT monitoring with deep learning algorithm-based Edge Image Processing Architecture	Raspberry Pi Camera with 5MP sensor	**• **Plant growth **• **Environment and Water quality	*-*	Wan et al. (2022)
GrowBot	Robotic system with U-Net: CNN	OV5647 CMOS image sensor with Raspberry Pi4	Plant growth based on nutrient deficiency and temperature stress	*Ocimum basilicum* (basil)	Bose and Hautop Lund (2022)
AscTec Navigator 3.4.5	UAV with built-in GPS	AscTec Falcon 8 octocopter (Ascending technologies, Germany) Sony α6000 24.3 MP camera with 20mm f/2.8 lens	**• **Leaf Area Index at 20m altitude **• **Leaf/biomass growth **• **Vegetation indices **• **Chlorophyll index	*A. hypogaea* L. (peanut)	Sarkar et al. (2021)
WEKA (Waikato Environment for Knowledge Analysis) software v3.8.4	ANN				
WOFOST	UAV imaging integration	-	Leaf area index (LAI), biomass, yield	*T. aestivum* (winter wheat)	Yang et al. (2021a)
Hyperspectral Reflectance	MLP, SVM and RF with remote sensing	UniSpec-DC Spectral Analysis System (PP Systems International Inc., USA)	**• **Biomass yield **• **Plant growth and development stages	*G. max* (soybean)	Yoosefzadeh-Najafabadi et al. (2021)
Greenotyper	U-Net: CNNs	RPi3 Model B with RPi Camera module v2.1	**• **Plant area **• **Greenness **• **Overlapping growth patterns	*Trifolium repens* (white clover)	Tausen et al. (2020)
Keras	U-Net based CNN segmentation model	2592 x 1944 x 3 resolution camera (5 MP)	Powdery mildew disease detection	*Cucumis sativus* (cucumber)	Lin et al. (2019)
CropDeep	RetNet with ResNet50 CNN	IoT cameras, Autonomous Spray robots, Autonomous Picking Robots, Mobicamera and Smartphone camera	**• **Precision farming **• **Plant identification, growth and location **• **Different plant variety monitoring **• **Fruit and vegetable health status	25 plant varieties including *L. sativa Linn.* (lettuce), *A. graveliens Linn.* (celery), *Cucumis Linn.* (cucumber), *B. oleracea Linn.* (cabbage), *S. oleracea Linn.* (spinach), *L. esculentum Mill.* (tomato), *R. sativus Linn.* (turnip)	Zheng et al. (2019)
Alexnet	CNN-Long-Short Term Memories (LSTM) architecture	Canon EOS 650D	Plant growth pattern of different genotypes	*A. thaliana*	Taghavi Namin et al. (2018)
Persistent Homology based topological methods	DIRT (Digital Imaging of Root Traits) Gaussian kernel density estimator Elliptical Fourier descriptors	-	**• **Leaf shape, serrations and root architecture **• **Discrimination between genotypes	*Solanum pennellii* (wild tomato)	Li et al. (2018)
PlantCV	U-Net based CNN	Raspberry Pi Camera	Plant convex hull, width and length	*A. thaliana*	Tovar et al. (2018)
		Nikon COOLPIX L830 Camera	Seed size, shape, count and color	*Chenopodium quinoa* Willd. (Quinoa)	
*LeafNet*	Caffe framework based Deep Learning CNN	*LeafSnap*, *Flavia* and *Foliage* dataset images using Mobile cameras (iPhones mostly)	Species identification through leaf features like edges and venations	*LeafSnap*, *Flavia* and *Foliage* dataset	Barré et al. (2017)
Deep Plant Phenomics (DPP)	Deep CNN with PlantCV module	Canon PowerShot SD1000 7 MP camera, Model B with Raspberry Pi 5 MP camera module	Leaf size, shape and leaf count	*A. thaliana* *N. tabacum* (tobacco)	Ubbens and Stavness (2017) Minervini et al. (2015)
*pheno*Seeder	KR 10 scara R600-Z300 robot (KUKA Roboter GmbH, Germany)	Oscar F-810C Camera (Allied-Vision Technologies, GmbH, Germany)	Seed projected area, length, width and color	*B. napus* (rapeseed), *H. vulgare* (barley) and *A. thaliana*	Jahnke et al. (2016)
		Grasshopper GRAS-50S5M-C Camera (Point Grey, Canada) with 35mm lens	Seed volume		
UAV remote sensing SAMPLINGTSPN	UAV and GPML (Gausian Processes for Machine Learning) Toolbox	MikroKopter, Hexa XL with Multispectral Tetracam Camera	Nitrogen level prediction at 30m altitude	*Z. mays* (maize)	Tokekar et al. (2016)
DIRT (Digital Imaging of Root Traits)	-	-	Root angles (top and bottom), stem diameter, width of root system	*Z. mays* (maize)	Das et al. (2015)
GARNICS	Robotic system with ML-based algorithms	Robot head with 4 x Point Grey Grasshopper, 3.45 μm pixels Camera and Schneider KreuznachXenoplan 1.4/17-0903 lenses Canon PowerShot SD1000 7 MP camera, Model B with Raspberry Pi 5 MP camera module	**• **Plant detection and localization **• **Plant and leaf segmentation **• **Leaf shade, appearance and difference detection **• **Leaf counting **• **Leaf growth tracking **• **Classification based on mutant and treatment recognition and age regression	*A. thaliana* *N. tabacum* (tobacco)	Minervini et al. (2015)

### 4.5 ML approaches in cell suspension cultures and bioreactors

Plant cell suspension cultures offer a unique platform for the production of recombinant proteins due to their ability to perform post-translational modifications similar to mammalian cells (Gutierrez-valdes et al., 2020). Plant cell suspension cultures are usually prepared from callus tissue in shaker flasks or fermenters to form single cells and small aggregates and growing plant cells in a liquid medium in a controlled environment, such as bioreactor, where various factors like temperature, pH, and ratio of nutrient are to be optimized for cell growth and protein production (Cardon et al., 2019). Several proteins have been produced in bioreactor using cell suspension cultures including ORF8, an accessory protein of SARS-CoV2 in suspension cultured tobacco BY-2 cells (Imamura et al., 2021), rrBChE, rice recombinant butyrylcholinesterase in rice cell suspension culture (Macharoen et al., 2021), LBT-Syn protein in carrot cell suspension culture (Carreño-Campos et al., 2022), taliglucerase (ELELYSO), a recombinant version of human glucocerebrosidase in carrot cell cultures (Mor, 2015) etc.

Large scale production of plant-expressed recombinant proteins can be achieved by growing the transformed plant cell in different bioreactor shapes, however, there are diverse problems to be addressed such as pH of media, minerals, growth regulators, cell density, gaseous atmosphere, agitation system and sterilization conditions (Ruffoni et al., 2010).

Now-a-days AI techniques are increasingly being applied to bioreactors, which are essential tools in bioprocessing for the production of various biological products such as recombinant proteins, vaccines, and biofuels. ML models can identify the optimal operating conditions, such as temperature, pH, dissolved oxygen, and nutrient concentrations, to maximize product yield and quality. By integrating with sensors, data acquisition systems and control algorithms, AI models can analyze data in real time and automatically adjust process parameter accordingly. AI can adapt and adjust process parameters for optimal performance, reducing the need for manual intervention.

Optimizing plant tissue culture media is a complicated and time-consuming process, which is influenced by genotype, mineral nutrients, plant growth regulators, vitamins and other factors. ML approaches such as multilayer perceptron neural network (MLPNN), k-nearest neighbors (KNN) and gene expression programming (GEP) were used for developing prediction models in optimizing plant tissue culture media composition (Hosseini et al., 2022). In another work, three ANN models: CIPnet, CWnet and DCnet were developed to predict the best media composition for callus weight (CW), callus induction percentage (CIP) and days to callus initiation (DC). The performance was satisfactory and showed the R^2^ values of 0.95, 0.95 and 0.88 for CIPnet, CW, and DCnet respectively (Munasinghe et al., 2020). The formation of foam in bioreactor is another major issue in pharmaceutical industry and creates operational issues. To address the issue in bioreactor, a CNN-based model was developed for the real-time identification of foam formation (Austerjost et al., 2021). Cell proliferation could be monitored through ML based algorithms. An ML model was trained for monitoring insect cell proliferation and viability percentage upon baculovirus infection in the bioreactor (Altenburg et al., 2023).

ANN based ML algorithm was used to control the micro-aerobic conditions to achieve a satisfactory product yield. Metabolic flux-based control strategy technique (SUPERSYS_MCU) was used to address the issue. To generate a surrogate model in the form of an ANN, the control strategy used simulations of a genome-scale metabolic model. The meta-model provided setpoints to the controller, allowing adjustment of the inlet airflow to control oxygen uptake rate (Zangirolami et al., 2021). Application of ANN models in predicting the system performance of osmotic membrane bioreactors (OMBRs) was investigated and such models developed showed good performance for the prediction of water flux and membrane fouling simulations (Viet and Jang, 2021).

Deep learning techniques in a hybrid semi metric modelling contest, such as deep feed forward neural network with varying depths, the rectified linear unit (ReLU) activation function, dropout regularization of network weights, and stochastic training with the ADAM method were explored (Mestre et al., 2022). Performance of ML algorithms was analyzed to predict n-caproate and n-caprylate productivities in bacteria using 16S rRNA amplicons in a bioreactor. The bioreactor performance was analyzed quantitatively and accurately from the dataset generated from different bioreactors. ML models were trained independently and tested with 16S rRNA amplicon sequencing data to predict n-caproate and n-caprylate productivities. The tests concluded that random forest was the best algorithm producing more consistent results with low error rate and more than 90% accuracy in the prediction of n-caproate and n-caprylate (Liu et al., 2022a). To predict the accuracy of real-time liquid level four ML algorithms, multiple linear regression (MLR), artificial neural network (ANN), random forest (RF), and support vector machine (SVM) with radial basis kernel were analyzed and found that ANN and RF models performed well (Yu et al., 2022).

### 4.6 AI in downstream processing

The market demand of biopharmaceutical products is constantly increasing every year and there is an increasing pressure on price reduction for global access to biological drugs. In order to meet the market demand, significant improvement has been carried out in upstream processes, however the productivity in downstream has not increased accordingly (Ötes et al., 2017). The most challenging phase of therapeutic protein production in industries is the downstream processing (DSP) and DSP is accounting for a large portion of the total production costs. The growing demand and developments in upstream processing of therapeutics have burdened the downstream purification processes, due to high cost and insufficient processing capacity (Li et al., 2019). DSP of recombinant therapeutic proteins involves a series of operation such as filtration, followed by capture, purification, and polishing steps mainly done by chromatography (Gaughan, 2016). Chromatography is considered as the workhorse of DSP because it can selectively enrich the target proteins while eliminating impurities and this is achieved by exploiting differences in molecular properties, such as size, charge and hydrophobicity (Bernau et al., 2022). The development of product specific chromatography-based purification techniques is time consuming and expensive because target proteins make up a small portion of the total protein in the initial plant extract. To address this issue, Buyel and Fischer (2014) created a general downstream procedure for the purification of recombinant proteins produced in plants with diverse features. This was done by concentrating on the resin’s ability to bind tobacco host cell proteins (HCPs) under various conditions such as pH and conductivity.

Recent developments in ML and DL based programs can be utilized to overcome the challenges in downstream processing (Bernau et al., 2022). ML has been applied to chromatography system to monitor real time processing, process optimization, retention time prediction and peak monitoring. In order to predict the chromatographic conditions (i.e., solvents and solvent ratio), three vectorization types such as learned embedding, extended-connectivity fingerprints (ECFP), ECFP encoder+FFNN and three machine learning approaches (FFNN, LSTM and CNN), DNN architectures and a set of hyperparameter values were investigated. The best results were achieved for the prediction of solvents and solvent ratio with ECFP LSTM auto-encoder with FFNN as the supervised machine-learning method with an accuracy of 0.95 for first task and 0.982 for second task respectively (Vaškevičius et al., 2021). Several ML models have been developed so far to address some of the challenges in downstream processing such as XGboost for the prediction of column performance (Jiang et al., 2022b), PeakBot for chromatographic peak prediction (Bueschl et al., 2022), DeepRT for peptide retention time prediction (Ma et al., 2017) and an algorithm to predict the HCPs elution behavior (Buyel et al., 2013).

## 5 Challenges and current limitations

Plant-based expression systems have several advantages for producing proteins, however, also come with limitations and challenges. Here are few limitations and challenges in plant-based expression systems such as low productivity, post-translational modification, protein stability, biosafety concerns, high costs of downstream processing, regulatory approval, and slow translation to applications (Schillberg et al., 2019; Schillberg and Finnern, 2021; Sethi et al., 2021). Even though the plant expression system is cheaper and more scalable than conventional expression systems, expression yields and appropriate post-translational modifications along the plant secretory pathway remain a challenge for many proteins. For instance, fusion viral glycoproteins often expressed in plants give low yield and may not be properly processed in some cases (Margolin et al., 2020b). In comparison to mammalian systems, plant-based expression systems introduce different glycosylation patterns which could have an effect on the immunogenicity and functionality of proteins. Although difficult, methods for achieving human-like glycosylation patterns in plants are being explored by engineering host systems using CRISPR/Cas9-based technologies. The intellectual property (IP) and regulatory body approval is one of the main hurdles in the adoption of molecular farming compared to commercial microbial and mammalian cell expression systems which have a proven track record, particularly in the field of biopharmaceutical manufacture. As a result, the industry continues to view molecular farming as risky and chooses to depend on its tried-and-true systems in most circumstances (Schillberg and Finnern, 2021). The possible hazards posed by genetically modified (GM) plants or animals, including the effect on biodiversity, ecological interactions, and possibility of unforeseen effects, must be carefully evaluated. There is a risk that the transgenes may unintentionally spread to other organisms through gene flow, such as cross-pollination or horizontal gene transfer. For molecular pharming processes and products to be safe, it is crucial to implement effective containment strategies, risk assessment and mitigation measures. Techniques such as chloroplast expression and transient expression in closed culture systems could circumvent the environmental risk of transgene transmission through pollen (Moon et al., 2019; Feng et al., 2022b).

**Figure 5:**
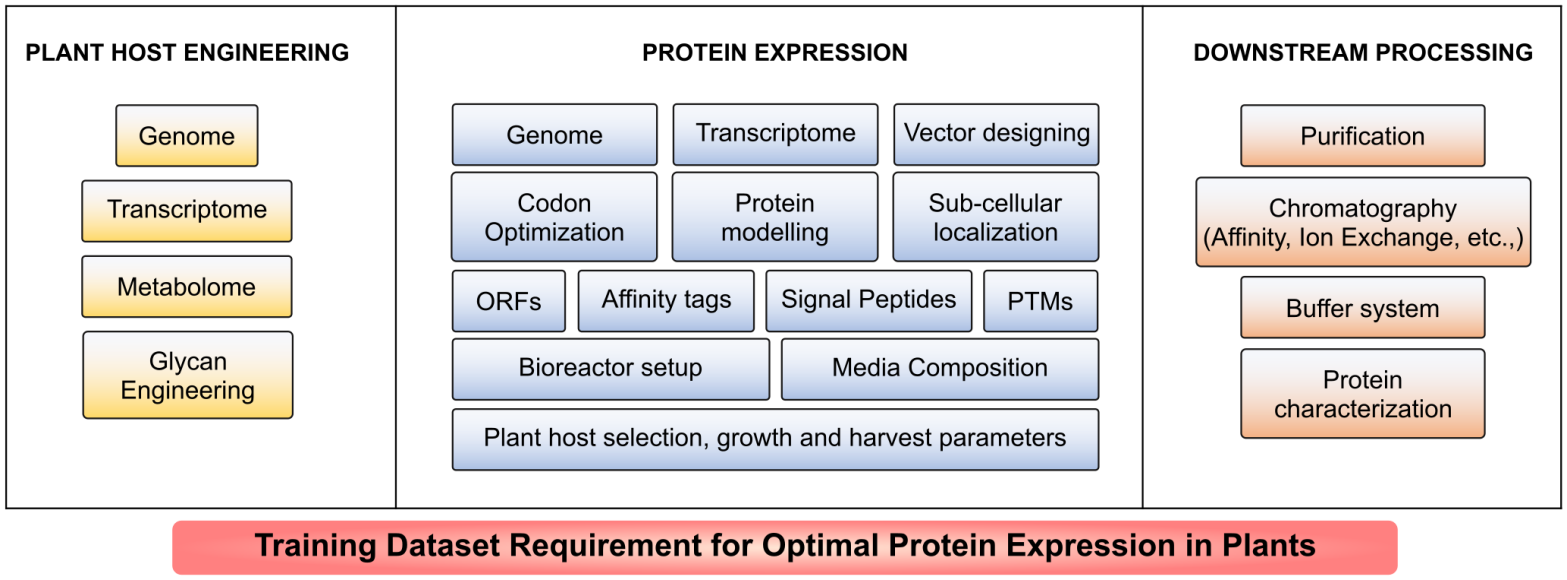
Training dataset requirement for optimal protein expression in plants. A large volume of data is required for prediction of optimum conditions at each stage including host engineering, expression and downstream processing for a specific protein to be expressed large-scale in plants.

AI-based tools have been developed and deployed for various microbial expression systems such as *E. coli*, *P. pastoris*, *S. cerevisiae* and mammalian cell expression systems including CHO, HEK293, HeLa and MCF7 (Linder et al., 2020; Van Brempt et al., 2020; Smiatek et al., 2021; Feng et al., 2022a; Li et al., 2022a; Packiam et al., 2022). Plant host system remains an unexplored arena for AI incorporation. Creation and maintenance of AI-based training models is mainly hindered by lack of abundant experimental dataset that include but not limited to genome, transcriptome and metabolome sequences; plant cell culture, plant growth and bioreactor conditions; protein extraction and optimization, purification strategies and relative parameters such as protein localization, structure, stability, catalytic activity and solubility. Such limited training dataset renders the ML approaches overfitting (Feng et al., 2020; van Dijk et al., 2021). Intervention of automation and AI models discussed in **Tables 1**, **2** to predict the conditions and maintenance for the large-scale production in plants is yet to be established as illustrated in **Figure 4**. Data integration of multiple parameters discussed in **Table 1** is needed for optimal protein expression. Further the generation of training dataset for plant cell culture condition optimization necessitates a large collection of data (van Dijk et al., 2021); and *in vitro* testing of enormous experimental procedures in different test conditions for an individual recombinant protein production in real-time is laborious; time-consuming; requires well-equipped research facility and investment for growth optimization, plant maintenance and downstream processing (Schillberg et al., 2019; Hesami et al., 2020; Sarker, 2021; van Dijk et al., 2021; Packiam et al., 2022). Even with the available omics data of model plants used in recombinant biologics production, expression training datasets are insufficient for AI-based host engineering and host selection, vector and gene designing, protein modelling, solubility and stability prediction as they are not integrated yet (van Dijk et al., 2021). A large number of data for each parameter (more than 10,000 data points if required) is needed to perform as an effective training dataset (Barré et al., 2017; Hesami et al., 2020; LaFleur et al., 2022; Yang et al., 2023). The illustration in **Figure 5** highlights the requirement of training datasets available globally that could build a web of AI-based prediction and optimization tools to tackle the challenges and increase the production of highly active next generation biologics. Several algorithms have been under-utilized or unutilized to increase the recombinant protein yield. ML algorithm could predict the signal peptides and increase the ER translocation rates in CHO cells (O’Neill et al., 2023), and yet not used in exploring recombinant biologics production in plants. CNN-based prediction models have been used effectively for increased protein expression in microbial systems (Zrimec et al., 2020) and so far no tool has been adapted for plant-based expression systems.

## 6 Conclusion and future directions

Plant molecular pharming offers efficient alternate host systems for expression of recombinant biologics. Moreover, the system is robust and cost-effective compared to other hosts. In this review, the concepts of AI in systems engineering for improved production of recombinant biologics were discussed. Several prediction and optimization parameters are known to increase the yield in different expression hosts and integration of machine learning algorithms is new to the plant molecular pharming field. Such plant-based expression parameters include host engineering, growth and maintenance, protein model designing, glycosylation, sialylation, epitope prediction, antibody identification& optimization, regulatory element prediction & optimization and protein stability and activity. Neural network-based ML models when integrated with systems engineering approaches could be advantageous during the manufacture of humanized forms of biologics at various stages of production including seed selection, germination, plant growth parameter optimization, monitoring, recombinant protein modelling, expression, extraction, purification and downstream processing. GEMs and other omics data availability favor the process of designing and optimization of protein production yet more omics (genomics, proteomics, transcriptomics and metabolomics) based studies are needed for complete utilization of ML tools. Transcriptome and metabolome profiles of specific plant hosts in the form of large training data sets need to be fed into neural networks, which then can be used to test the desired function (such as gene knock-out or knock-in). Similarly, parameters of protein production solely based on plant system are to be created as codes using language models and integrated as hierarchical architectures using neural networks. Datasets trained with the discussed parameters using ML models for protein expression in plants could aid in an effective modelling of recombinant biologics and prediction of accurate conditions for protein expression in different plant hosts including but not limited to *N. benthamiana*, *N. tabacum*, *L. sativa* and *O. sativa*. Such ML-based techniques will reduce the time frame and cost of reagents in all the levels of plant-based biologics production rendering functional and active products.

## Author contributions

RS proposed the idea of application of AI in plant molecular pharming; SP designed the review manuscript. TG drafted systems biology; SP and TV drafted AI integration concepts and improvised systems biology concepts. RS and BS revised and corrected the manuscript. AS gave expert comments on the technical aspects. All authors contributed to the article and approved the submitted version.
